# Supplementary material for: Causal Relationship between Aging and Anorexia Nervosa: A White-Matter-Microstructure-Mediated Mendelian Randomization Analysis
Source: Biomedicines. 2024 Aug 16;12(8):1874. doi: 10.3390/biomedicines12081874 (PMC11351342; doi:10.3390/biomedicines12081874)
Supplement: Supplementary file 1 [file biomedicines-12-01874-s001.zip › Supplementary Figure/Figure S8/Leaveoneout plot of MR analysis for TL to ubm-b-2077.pdf]

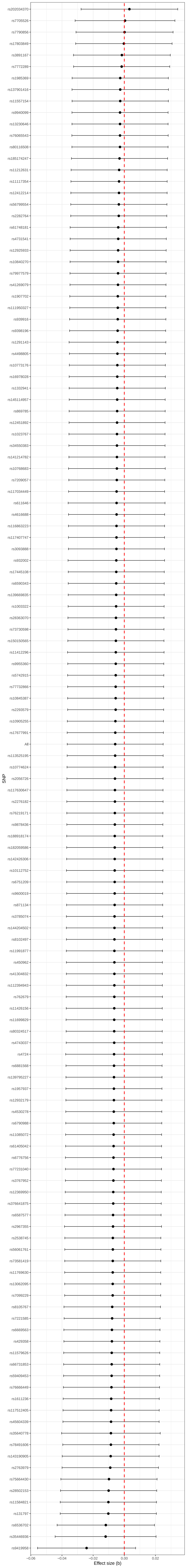
rs202034370
rs7705526
rs7790856
rs17803849
rs3891167
rs7772289
rs1985369
rs137901416
rs11557154
rs9940099
rs13230646
rs76065543
rs80116508
rs185174247
rs11212631
rs11117354
rs12412214
rs56799554
rs2282764
rs61748181
rs4731541
rs12925933
rs10840270
rs79977579
rs41269079
rs1907702
rs111950327
rs939916
rs9398196
rs1291143
rs4498805
rs10773176
rs16978028
rs1332941
rs145114957
rs869785
rs12451892
rs1023767
rs34550383
rs141214782
rs10768683
rs7209057
rs117034449
rs611646
rs4616688
rs116863223
rs117407747
rs3093888
rs932002
rs17445108
rs6590343
rs139669835
rs1003322
rs28363070
rs73730598
rs150150565
rs11412296
rs9955360
rs5742915
rs77732866
rs10845387
rs2293579
rs10905255
rs17677991
All
rs113525195
rs10774624
rs2056726
rs117630647
rs2276182
rs76219171
rs9878436
rs188918174
rs182059586
rs142426306
rs10112752
rs6751209
rs9600019
rs871134
rs3785074
rs144204502
rs8102497
rs11991877
rs450962
rs41304832
rs112394943
rs762679
rs11426156
rs11699829
rs80324517
rs4743037
rs4724
rs6881568
rs139795227
rs1957937
rs12932179
rs4530278
rs6790988
rs11085072
rs61405042
rs6776756
rs77231040
rs3767952
rs12369950
rs376641875
rs6587577
rs2967355
rs2538745
rs56061761
rs73581419
rs11769630
rs13062095
rs7099229
rs8105767
rs7221585
rs6669563
rs429358
rs11579626
rs66731853
rs59409453
rs76666449
rs1611236
rs117512405
rs45604339
rs35640778
rs78491606
rs143190905
rs2763979
rs75664430
rs28502153
rs11584821
rs131797
rs6536702
rs35446936
rs9419958
SNP
−0.06
−0.04
−0.02
0.00
0.02
Effect size (b)
